# Experience With More Than 1,000 Cuffed Tunneled Hemodialysis Catheter Insertions Over Four Years at a Single Center

**DOI:** 10.7759/cureus.111073

**Published:** 2026-06-18

**Authors:** Maulik A Patel, Pranjal Kashiv, Khushboo Saxena, Manish Balwani, Vivek Kute

**Affiliations:** 1 Nephrology, Shankus Hospital, Mehsana, IND; 2 Nephrology, All India Institute of Medical Sciences, Nagpur, IND; 3 Nephrology, Institute of Kidney Diseases and Research Centre (IKDRC), Ahmedabad, IND; 4 Nephrology, Saraswati Kidney Care Center, Nagpur, IND; 5 Nephrology, Jawaharlal Nehru Medical College, Wardha, IND

**Keywords:** end-stage renal disease (esrd), hemodialysis vascular access, internal jugular vein catheterization, interventional nephrology, tunneled cuffed catheter

## Abstract

Introduction: Cuffed tunneled hemodialysis catheters are an important vascular access option for patients with end-stage renal disease (ESRD) when arteriovenous fistula (AVF) creation is not feasible or when urgent initiation of hemodialysis is required. However, clinical outcomes may vary according to the catheter insertion site.

Objective: To compare procedural safety, biochemical outcomes, and six-month clinical outcomes among different tunneled hemodialysis catheter insertion sites.

Methods: This retrospective observational study included 1,050 consecutive patients who underwent tunneled hemodialysis catheter insertion over four years at a tertiary care center. Patients were categorized according to catheter insertion site into right internal jugular vein (RIJV; *n* = 535), left internal jugular vein (LIJV; *n *= 416), subclavian vein (*n *= 53), and femoral vein (*n *= 46) groups. Baseline characteristics, procedure-related complications, laboratory outcomes at three months, and clinical outcomes at six months were compared using appropriate statistical analyses.

Results: Baseline demographic and laboratory characteristics were comparable among all groups (all *P *> 0.05). Procedure-related complications were significantly more frequent in the subclavian and femoral groups, including exit-site bleeding (*P *= 0.032), catheter malposition (*P *= 0.028), hematoma formation (*P *= 0.018), arterial puncture (*P *= 0.022), hypoxia (*P *= 0.036), arrhythmia (*P *= 0.041), and pneumothorax (*P *= 0.048). At three months, patients with subclavian and femoral access demonstrated significantly poorer biochemical profiles, characterized by lower hemoglobin and serum albumin levels and higher leukocyte counts, serum creatinine, C-reactive protein, phosphorus, and intact parathyroid hormone levels (all p<0.05). At six months, internal jugular vein access was associated with significantly higher rates of successful AVF creation (*P *= 0.018) and ongoing catheter survival (*P *= 0.012). Conversely, subclavian and femoral access were associated with significantly higher rates of catheter dysfunction (*P *= 0.015), catheter-related bloodstream infection (*P *= 0.013), and mortality (*P *= 0.020).

Conclusions: Internal jugular vein access, particularly RIJV access, was associated with superior procedural safety, more favorable biochemical profiles, and better six-month clinical outcomes compared with subclavian and femoral access. These findings support the preferential use of internal jugular vein access for tunneled hemodialysis catheter placement whenever feasible.

## Introduction

End-stage renal disease (ESRD) remains a major global health burden, with the prevalence and mortality of chronic kidney disease continuing to rise worldwide. Diabetes mellitus is one of the leading causes of advanced kidney disease, and timely management of risk factors is essential to delay disease progression and reduce the risk of kidney failure [1-6]. For patients requiring maintenance hemodialysis, the arteriovenous fistula (AVF) remains the preferred long-term vascular access because of its superior patency and lower risk of infection compared with central venous catheters and vascular grafts [7-8].

Despite these recommendations, many patients initiate hemodialysis using a catheter because of late referral, urgent dialysis requirements, inadequate peripheral vasculature, or delayed vascular access planning [1-2,9-10]. When catheter use is required beyond a short period, tunneled cuffed hemodialysis catheters provide greater stability and a lower risk of infection than non-tunneled catheters. They often serve as a bridge to AVF creation or, in selected patients, as a longer-term vascular access option [7-8,10-11].

The choice of catheter insertion site is an important determinant of procedural success and long-term outcomes. The right internal jugular vein (RIJV) is generally preferred because of its relatively straight course to the superior vena cava and lower risk of central venous stenosis. In contrast, subclavian vein access is generally discouraged because of the risk of venous stenosis, whereas femoral vein access is typically reserved for patients in whom upper-body access is not feasible [7-8,12-23].

The use of ultrasound and fluoroscopic guidance, together with standardized catheter insertion techniques, has further improved procedural safety and technical success rates [10,24-26]. Although several studies have evaluated outcomes associated with tunneled hemodialysis catheters, real-world data from high-volume centers, particularly in resource-limited settings, remain limited. Direct comparisons of outcomes across different catheter insertion sites are especially scarce [13-14,19-20,27-30]. Recent studies have demonstrated that ultrasound-guided catheter placement can be performed safely with or without fluoroscopic assistance, that alternative access routes such as the external jugular vein may be useful when right internal jugular access is unavailable, and that catheter dysfunction may be influenced by catheter tip design and positioning [28,31].

Therefore, this study evaluated 1,050 consecutive cuffed tunneled hemodialysis catheter insertions performed over a four-year period. The primary objective was to compare six-month clinical outcomes across catheter insertion sites; secondary objectives were to compare procedural safety and three-month biochemical outcomes

## Materials and methods

This retrospective observational study was conducted in the Department of Nephrology at Shankus Hospital, Mehsana, Gujarat, India. A total of 1,050 consecutive tunneled cuffed hemodialysis catheter insertions performed between November 2021 and October 2025 were included in the analysis. The study was approved by the Institutional Ethics Committee (IEC Registration No. ECR/1615/Inst/GJ/2021; Approval No. 27, dated April 11, 2026). Written informed consent for both the procedure and publication was obtained from all patients or their legally authorized representatives.

Patients with ESRD who required tunneled hemodialysis catheter placement were eligible for inclusion. All consecutive eligible patients undergoing tunneled cuffed hemodialysis catheter insertion during the study period were included; non-tunneled (temporary) catheter insertions were not analyzed, and no additional exclusion criteria were applied. Based on the catheter insertion site, patients were categorized into four groups: RIJV, left internal jugular vein (LIJV), subclavian vein, and femoral vein. This classification enabled comparison of procedural safety, biochemical outcomes, and clinical outcomes across different vascular access sites. Because catheter site selection was not randomized and was determined according to clinical and anatomical considerations, the potential for selection bias cannot be excluded.

All procedures were performed using 15-French cuffed tunneled hemodialysis catheters. Catheter length was selected according to the insertion site: 24 cm (tip-to-cuff) for RIJV access, 28 cm for LIJV and subclavian access, and 36 cm for femoral access. All catheter insertions were performed in a dedicated catheterization laboratory by an experienced nephrologist trained in interventional procedures.

Real-time ultrasound guidance was used for venous cannulation to improve procedural accuracy and minimize complications. In patients with a pre-existing temporary internal jugular catheter, catheter exchange was performed using an over-the-guidewire technique. Following successful venous access, local anesthesia was administered, a subcutaneous tunnel was created, serial dilatation was performed, and the catheter was advanced under fluoroscopic guidance. For internal jugular and subclavian access, correct tip position was confirmed at the cavoatrial junction or within the right atrium; for femoral access, the catheter was advanced to achieve the most cranial inferior vena cava or cavoatrial position permitted by catheter length. After the procedure, each catheter was flushed and locked with heparin solution. Post-procedural chest radiography was performed to confirm appropriate catheter placement and to exclude immediate complications.

Demographic characteristics, comorbid conditions, and catheter insertion sites were recorded for all patients. Procedural variables included de novo catheter insertion versus over-the-guidewire exchange, procedure-related complications, and the requirement for post-procedural imaging. Laboratory parameters were assessed at baseline and at three months and included hemoglobin, total leukocyte count, platelet count, serum electrolytes, blood urea, serum creatinine, serum albumin, C-reactive protein (CRP), venous bicarbonate, calcium, phosphorus, intact parathyroid hormone (iPTH), uric acid, and iron profile parameters.

Clinical outcomes were evaluated at six months and categorized into mutually exclusive endpoints according to the patient's predominant clinical status: successful transition to an AVF or vascular graft with catheter removal, continued hemodialysis through a functioning catheter, catheter removal due to dysfunction or thrombosis, catheter removal due to catheter-related bacteremia, and all-cause mortality. Catheter dysfunction was defined as catheter removal due to inadequate blood flow, thrombosis, or fibrin sheath formation. Catheter-related bacteremia was defined as catheter removal due to bloodstream infection without another identifiable source.

Because this retrospective study included all consecutive eligible patients during the study period, a formal sample size calculation was not performed. Analyses for each variable were based on the available data at the respective time points. Statistical analyses were conducted using SPSS version 25.0 (IBM Corp., Armonk, NY). Continuous variables are presented as mean ± standard deviation (SD) and were compared using one-way analysis of variance (ANOVA). Categorical variables are presented as frequencies and percentages and were compared using the chi-square test or Fisher’s exact test, as appropriate. All statistical tests were two-tailed, and a *P*-value of <0.05 was considered statistically significant. The study was conducted in accordance with the principles of the Declaration of Helsinki and was reported in compliance with the Strengthening the Reporting of Observational Studies in Epidemiology (STROBE) guidelines [32]. Six-month clinical outcomes were ascertained for all 1,050 patients, with no loss to follow-up for the primary clinical endpoints.

## Results

A total of 1,050 patients underwent insertion of cuffed tunneled hemodialysis catheters during the study period. The cohort included 575 men (54.8%) and 475 women (45.2%), with a mean age of 54.1 ± 13.2 years. Most procedures involved new venous cannulation under ultrasound guidance (*n* = 854, 81.3%), whereas 196 (18.7%) were performed using an over-the-guidewire exchange technique. The RIJV was the most common access site (*n* = 535, 51.0%), followed by the LIJV (*n* = 416, 39.6%), subclavian vein (*n* = 53, 5.0%), and femoral vein (*n* = 46, 4.4%).

Baseline demographic and laboratory characteristics are presented in Table 1. The study groups were well matched at baseline, indicating a high degree of cohort homogeneity. No significant differences were observed in age, sex distribution, comorbidities, hematological parameters, renal function indices, inflammatory markers, electrolyte profile, mineral and bone metabolism parameters, or iron status (all *P* > 0.05). These findings suggest that subsequent differences in procedural, biochemical, and clinical outcomes were unlikely to be attributable to baseline imbalances, thereby strengthening the internal validity of the comparative analyses.

**Table 1:** Baseline demographic and clinical characteristics according to the catheter insertion site (n = 1,050). Data are presented as mean ± standard deviation (SD) or number (%). Continuous variables were compared using one-way analysis of variance (ANOVA), and categorical variables were compared using the chi-square test. A *P*-value <0.05 was considered statistically significant. IJV, internal jugular vein; BMI, body mass index; WBC, white blood cell count; CRP, C-reactive protein; TIBC, total iron-binding capacity.

Variable	Right IJV (*n *= 535)	Left IJV (*n *= 416)	Subclavian (*n *= 53)	Femoral (*n *= 46)	*P*-value	Test statistic
Age (years), mean ± SD	54.1 ± 13.0	55.4 ± 12.7	53.6 ± 13.4	54.8 ± 13.6	0.79	*F *= 0.62
Male sex, *n* (%)	292 (54.6)	231 (55.5)	30 (56.6)	25 (54.3)	0.96	*χ*² = 0.41
BMI (kg/m²), mean ± SD	23.5 ± 3.1	23.1 ± 3.4	23.6 ± 3.0	23.0 ± 3.5	0.84	*F *= 0.74
Diabetes mellitus, *n* (%)	208 (38.9)	157 (37.7)	21 (39.6)	17 (37.0)	0.95	*χ*² = 0.52
Hypertension, *n* (%)	379 (70.8)	299 (71.9)	38 (71.7)	32 (69.6)	0.93	*χ*² = 0.48
Coronary artery disease, *n* (%)	86 (16.1)	69 (16.6)	10 (18.9)	7 (15.2)	0.89	*χ*² = 0.67
Hemoglobin (g/dL), mean ± SD	10.5 ± 1.1	10.3 ± 1.3	10.2 ± 1.4	10.4 ± 1.2	0.61	*F *= 0.72
WBC (×10⁹/L), mean ± SD	6.6 ± 1.3	6.4 ± 1.5	6.7 ± 1.6	6.5 ± 1.4	0.66	*F *= 0.58
Platelet count (×10⁹/L), mean ± SD	166 ± 58	161 ± 64	159 ± 60	162 ± 63	0.73	*F *= 0.41
Sodium (mmol/L), mean ± SD	137.2 ± 3.1	136.4 ± 3.8	137.0 ± 3.5	136.6 ± 3.9	0.69	*F *= 0.63
Potassium (mmol/L), mean ± SD	4.5 ± 0.5	4.6 ± 0.6	4.4 ± 0.6	4.6 ± 0.5	0.58	*F *= 0.81
Urea (mg/dL), mean ± SD	109 ± 27	112 ± 31	111 ± 30	113 ± 29	0.77	*F *= 0.37
Serum creatinine (mg/dL), mean ± SD	8.4 ± 2.0	8.7 ± 2.3	8.5 ± 2.1	8.6 ± 2.2	0.74	*F *= 0.46
Serum albumin (g/dL), mean ± SD	3.5 ± 0.5	3.3 ± 0.6	3.4 ± 0.6	3.3 ± 0.5	0.62	*F *= 0.69
CRP (mg/L), mean ± SD	5.1 ± 1.9	5.4 ± 2.2	5.3 ± 2.3	5.6 ± 2.4	0.68	*F *= 0.54
Venous bicarbonate (mmol/L), mean ± SD	22.3 ± 2.6	22.0 ± 2.8	22.1 ± 2.9	21.8 ± 3.0	0.75	*F *= 0.43
Corrected calcium (mg/dL), mean ± SD	8.8 ± 0.6	8.6 ± 0.7	8.7 ± 0.6	8.5 ± 0.7	0.64	*F *= 0.66
Phosphorus (mg/dL), mean ± SD	5.4 ± 1.2	5.6 ± 1.3	5.5 ± 1.2	5.7 ± 1.4	0.66	*F *= 0.57
Intact parathyroid hormone (pg/mL), mean ± SD	315 ± 145	335 ± 165	325 ± 155	340 ± 170	0.79	*F *= 0.31
Uric acid (mg/dL), mean ± SD	6.2 ± 1.4	6.5 ± 1.6	6.4 ± 1.7	6.6 ± 1.8	0.65	*F *= 0.61
Serum iron (µg/dL), mean ± SD	72 ± 22	70 ± 24	74 ± 25	71 ± 23	0.72	*F *= 0.48
TIBC (µg/dL), mean ± SD	265 ± 60	260 ± 65	268 ± 62	262 ± 64	0.68	*F *= 0.53
Serum ferritin (ng/mL), mean ± SD	410 ± 170	430 ± 180	420 ± 190	435 ± 185	0.75	*F *= 0.39

Procedure-related complications

Procedure-related complications by catheter insertion site are presented in Table 2. Complications were more frequent among patients who underwent subclavian or femoral catheter placement than among those who received internal jugular vein access. Exit-site bleeding was the most common complication observed, while non-jugular access was also associated with higher rates of catheter malposition, arrhythmia, hypoxia, arterial puncture, hematoma formation, and pneumothorax. Overall, these findings indicate a superior procedural safety profile for internal jugular vein access compared with subclavian and femoral access.

**Table 2:** Procedure-related complications according to the catheter insertion site. Data are presented as number (%). Categorical variables were compared using the chi-square test or Fisher's exact test, as appropriate. A *P*-value <0.05 was considered statistically significant. *Fisher's exact test was applied because of sparse event counts. IJV, internal jugular vein

Complication, *n* (%)	Right IJV (*n* = 535)	Left IJV (*n* = 416)	Subclavian (*n* = 53)	Femoral (*n* = 46)	*P*-value	Test statistic
Exit-site bleeding	60 (11.2)	48 (11.5)	10 (18.9)	9 (19.6)	0.032	*χ*² = 8.74
Catheter malposition	9 (1.7)	7 (1.7)	4 (7.5)	3 (6.5)	0.028	*χ*² = 7.03
Arrhythmia	6 (1.1)	5 (1.2)	3 (5.7)	2 (4.3)	0.041	*χ*² = 6.42
Hypoxia	7 (1.3)	6 (1.4)	4 (7.5)	3 (6.5)	0.036	*χ*² = 6.71
Arterial puncture	5 (0.9)	4 (1.0)	3 (5.7)	2 (4.3)	0.022	*χ*² = 7.38
Hematoma formation	8 (1.5)	6 (1.4)	5 (9.4)	4 (8.7)	0.018	*χ*² = 9.11
Pneumothorax*	3 (0.6)	2 (0.5)	2 (3.8)	0 (0.0)	0.048	Fisher's exact test

Laboratory outcomes at three months

Laboratory outcomes at three months according to catheter insertion site are presented in Table 3. Patients in the subclavian and femoral groups demonstrated consistently poorer biochemical profiles than those in the internal jugular vein groups. Specifically, they had lower hemoglobin and platelet counts, higher leukocyte counts, more pronounced electrolyte abnormalities, impaired renal function, elevated CRP levels, and greater disturbances in mineral and bone metabolism parameters. Additionally, lower serum iron and total iron-binding capacity (TIBC) levels, together with higher ferritin concentrations, suggested the presence of inflammation-associated functional iron deficiency in patients with non-jugular catheter access.

**Table 3:** Laboratory parameters at three-month follow-up according to the catheter insertion site. Data are presented as mean ± standard deviation (SD). Continuous variables were compared using one-way analysis of variance (ANOVA). A *P*-value <0.05 was considered statistically significant. IJV, internal jugular vein; WBC, white blood cell count; CRP, C-reactive protein

Parameter	Right IJV (*n *= 535)	Left IJV (*n *= 416)	Subclavian (*n *= 53)	Femoral (*n *= 46)	*P*-value	*F*-value
Hemoglobin (g/dL)	10.6 ± 1.1	10.4 ± 1.2	9.4 ± 1.3	9.1 ± 1.4	0.028	3.24
WBC (×10⁹/L)	6.7 ± 1.4	6.8 ± 1.5	8.5 ± 2.0	9.0 ± 2.3	0.032	3.47
Platelet count (×10⁹/L)	168 ± 57	165 ± 60	140 ± 55	135 ± 52	0.024	3.12
Sodium (mmol/L)	137.5 ± 3.0	137.0 ± 3.2	134.5 ± 3.8	133.8 ± 4.0	0.041	2.86
Potassium (mmol/L)	4.6 ± 0.5	4.7 ± 0.6	5.2 ± 0.7	5.5 ± 0.8	0.018	4.03
Urea (mg/dL)	105 ± 26	107 ± 28	130 ± 32	138 ± 35	0.021	3.38
Serum creatinine (mg/dL)	8.0 ± 1.9	8.2 ± 2.0	9.3 ± 2.3	9.8 ± 2.5	0.026	3.15
Serum albumin (g/dL)	3.5 ± 0.5	3.4 ± 0.5	3.0 ± 0.6	2.8 ± 0.6	0.021	3.27
CRP (mg/L)	4.9 ± 1.8	5.1 ± 2.0	8.5 ± 2.8	9.2 ± 3.1	0.014	4.48
Venous bicarbonate (mmol/L)	22.5 ± 2.5	22.2 ± 2.7	20.5 ± 3.0	19.8 ± 3.2	0.037	2.97
Corrected calcium (mg/dL)	8.8 ± 0.6	8.7 ± 0.6	8.1 ± 0.7	7.8 ± 0.8	0.019	3.59
Phosphorus (mg/dL)	5.3 ± 1.1	5.4 ± 1.2	6.2 ± 1.4	6.6 ± 1.5	0.016	3.74
Intact parathyroid hormone (pg/mL)	330 ± 150	340 ± 160	420 ± 190	470 ± 210	0.023	3.31
Uric acid (mg/dL)	6.3 ± 1.4	6.5 ± 1.5	7.8 ± 1.9	8.3 ± 2.1	0.020	3.09
Serum iron (µg/dL)	70 ± 20	68 ± 22	52 ± 18	48 ± 16	0.034	2.81
Total iron-binding capacity (µg/dL)	260 ± 58	255 ± 60	220 ± 55	210 ± 50	0.028	3.04
Serum ferritin (ng/mL)	420 ± 180	440 ± 190	620 ± 250	680 ± 270	0.019	3.42

Six-month clinical outcomes

Six-month clinical outcomes according to catheter insertion site are presented in Table 4. Outcomes were generally more favorable among patients with internal jugular vein access than among those with subclavian or femoral access. Rates of successful AVF creation were substantially higher in the internal jugular groups, likely because preservation of alternative vascular sites facilitated subsequent AVF creation and maturation. Continued dialysis through a functioning catheter was also more common among patients with internal jugular access. In contrast, catheter removal due to dysfunction or catheter-related bacteremia occurred more frequently in the non-jugular groups, and mortality rates were markedly higher among patients with subclavian and femoral access. Outcomes were comparable between the right and left internal jugular groups across major clinical endpoints, supporting the use of left internal jugular access as a reasonable alternative when right-sided access is unavailable or contraindicated.

**Table 4:** Six-month clinical outcomes according to the catheter insertion site. Data are presented as number (%). Categorical variables were compared using the chi-square test or Fisher's exact test, as appropriate. A *P*-value <0.05 was considered statistically significant. IJV, internal jugular vein; AVF, arteriovenous fistula

Outcome	Right IJV (*n *= 535), *n* (%)	Left IJV (*n *= 416), *n* (%)	Subclavian (*n *= 53), *n* (%)	Femoral (*n *= 46), *n* (%)	*P*-value	Test statistic
Successful AVF creation (catheter removed)	253 (47.3)	197 (47.4)	5 (9.4)	3 (6.5)	0.018	*χ*² = 15.14
Ongoing dialysis with a functioning catheter	148 (27.7)	116 (27.9)	4 (7.5)	0 (0.0)	0.012	Fisher's exact test
Catheter removal due to dysfunction	45 (8.4)	35 (8.4)	14 (26.4)	13 (28.3)	0.015	*χ*² = 11.92
Catheter removal due to bacteremia	65 (12.1)	50 (12.0)	17 (32.1)	15 (32.6)	0.013	*χ*² = 10.68
Mortality	24 (4.5)	18 (4.3)	13 (24.5)	15 (32.6)	0.020	*χ*² = 13.37

## Discussion

In this large single-center cohort, catheter insertion site emerged as a major correlate of clinical outcomes. Although baseline characteristics were well balanced across the study groups, subclavian and femoral access were associated with higher rates of procedural complications, poorer biochemical profiles at three months, and less favorable six-month clinical outcomes compared with internal jugular access. These findings are consistent with current recommendations favoring RIJV access whenever feasible [7-8,10,12-14,19,22-23].

The lower complication burden associated with internal jugular access is biologically plausible and aligns with contemporary evidence demonstrating that image-guided catheter placement achieves high technical success rates with fewer mechanical complications [10,24-26]. The internal jugular route provides a relatively straight course to the cavoatrial junction, thereby reducing the risk of catheter malposition and vascular injury. In contrast, subclavian and femoral cannulation may be technically more challenging, particularly in patients with obesity, edema, previous vascular instrumentation, or anatomical limitations. These factors likely contributed to the higher rates of hematoma formation, arterial puncture, hypoxia, and pneumothorax observed in the non-jugular access groups [12-14,16,23].

Infectious complications remain a major limitation of catheter-based hemodialysis access. Previous studies have demonstrated that the risk of catheter-related bacteremia is influenced by both insertion site and catheter dwell time, with femoral and other non-jugular access routes generally associated with less favorable outcomes than internal jugular access [15-18,20,22,31]. In the present study, the higher frequency of catheter removal due to bacteremia among patients with subclavian and femoral access underscores the importance of meticulous aseptic technique, appropriate exit-site care, and timely transition to permanent vascular access whenever feasible [11,20,31]. These findings further support the preferential use of internal jugular access in centers managing large numbers of catheter-dependent hemodialysis patients.

Catheter dysfunction demonstrated a similar site-dependent pattern. Functional catheter failure is commonly related to thrombus formation, fibrin sheath development, inadequate blood flow, and catheter malposition [12,18,21,31,33-34]. The higher rates of catheter dysfunction observed with subclavian and femoral access in our cohort are consistent with previous observational studies and systematic reviews demonstrating that catheter design, tip position, and venous anatomy significantly influence long-term catheter patency [12,14,21,28,31,33-35]. In the femoral group, the 36 cm catheter length may, in some patients, have positioned the tip within the inferior vena cava rather than the right atrium; suboptimal tip position is a recognized risk factor for inferior vena cava stenosis, thrombosis, and catheter malfunction, and may have contributed to the higher rate of catheter dysfunction observed with femoral access [29,35]. These findings highlight the importance of evaluating long-term catheter performance in addition to immediate procedural success.

Another important observation was the less favorable biochemical profile observed at three months among patients with non-jugular catheters. Lower hemoglobin levels, higher inflammatory markers, and greater disturbances in mineral and bone metabolism may reflect reduced dialysis adequacy, increased inflammatory burden, or recurrent subclinical catheter-related complications [5-6,11,20,30]. Although relatively few studies have systematically evaluated biochemical outcomes according to catheter insertion site, our findings suggest that vascular access selection may influence not only mechanical outcomes but also the overall clinical stability of patients receiving maintenance hemodialysis. Consequently, access planning may have important downstream effects on anemia management, inflammation control, and mineral metabolism.

The mortality findings in our cohort are clinically relevant. Six-month mortality was substantially higher among patients with subclavian and femoral access than among those with internal jugular access. Although mortality in dialysis populations is influenced by multiple factors, including comorbid conditions, delayed referral, infection burden, and catheter dependence itself, the catheter insertion site may provide additional prognostic information [3,6,34]. The comparable outcomes observed between right and left internal jugular access are also clinically important, supporting the use of LIJV access as a reasonable alternative when right-sided access is unavailable or contraindicated [12,19,23]. This flexibility is particularly valuable in patients with previous right-sided instrumentation, central venous thrombosis, or challenging vascular anatomy.

Strengths and limitations

The strengths of this study include its large sample size, inclusion of consecutive patients, standardized catheter insertion technique, and comprehensive assessment of procedural, biochemical, and clinical outcomes. However, several limitations should be acknowledged. The retrospective single-center design may limit generalizability, and the non-randomized selection of catheter insertion sites introduces the possibility of selection bias. Multivariable and propensity-score analyses were not performed; accordingly, the observed differences should be interpreted as associations rather than independent effects of catheter insertion site. In addition, time-to-event analyses for catheter survival were not performed. Future multicenter prospective studies are needed to validate these findings and further clarify the impact of catheter insertion site on long-term outcomes.

From a clinical perspective, these findings support a hierarchical approach to tunneled hemodialysis catheter placement. The RIJV should remain the preferred access site; the LIJV represents an acceptable alternative, and subclavian or femoral access should be reserved for carefully selected patients in whom jugular access is not feasible [7-8,10,13-14,19,23]. Early referral and timely AVF planning remain essential to minimize prolonged catheter dependence and improve long-term outcomes [2-3,5-8].

## Conclusions

In this large single-center study of 1,050 patients, tunneled hemodialysis catheter insertion was safe when performed by experienced operators using ultrasound and fluoroscopic guidance. Nevertheless, the catheter insertion site remained a major determinant of patient outcomes. Internal jugular access, particularly RIJV access, was associated with lower procedural complication rates, more favorable biochemical profiles at three months, and superior six-month clinical outcomes compared with subclavian or femoral access. LIJV access remained a reasonable alternative when right-sided access was not feasible or was contraindicated.
